# The Fine Old Irish Surgeon*Reproduced by request.

**Published:** 1900-12

**Authors:** F. E. Daniel


### The Fine Old Irish Surgeon.*

BY F. E. DANIEL, M. D.

(Air: *"The Fine Old English Gentleman."*)

There was a fine old Irish surgeon, from Erin's emerald isle,
Who dashed around in a spanking double-team in the biggest kind of style:
His reputation as an operator extended far and wide,
And patients by the hundred flocked to see him in an ever swelling tide,
This fine old Irish surgeon, from Erin's emerald isle.

This fine old Irish surgeon was a man of wondrous guile,
Who by the practice of surgery and sundry other arts had managed to accumulate quite a respectable little pile;
He had a perfect mania for operating, and a fee he never missed;
For sufficient pecuniary consideration he'd tackle anything on God's green earth, from a bone fellon to a four-hundred-pound firmly adherent multilocular ovarian cyst,—
This fine old Irish surgeon, from Erin's emerald isle.

This fine old Irish surgeon, his leisure to beguile,
Would tell the darndest, toughest, most highly-improbable, Munchausen-like surgical yarns and never crack a smile;
He didn't care a continental for his patient's souls,—that was none of his business; for their bodies he cared still less,
And although most of his pataients died on the operating table, and the others immediately after being removed, he'd claim with more truth than poetry that every operation was a "bloody, big success,"
This fine old Irish surgeon, from Erin's emerald isle.

*Reproduced by request.

One day there came a woman whose baby, she related,
In consequence of a grain of corn firmly impacted in the trachea,
was well nigh asphyxiated,
Och, Doctor, dear, its dead, he is, as sure as ye's are born,
Unless be the holy mother ye's can extricate the murtherin' grain
of corn,
Ye's fine old Irish surgeon, from Erin's emerald isle.

Be aisy, now, the doctor said, and never ye's moind a whinnet;
A sample of me wondrous skill I'll show ye's in a minit,—
Tracheotomy, I'll perform, the sixteenth case today (moind that,
now),
And be the holy powers, while ye wait, that same pesky little grain
of corn I'll quickly bring away,
Said this fine old Irish surgeon, in his grandi-loquent style.

The baby then he quickly took,—now in *articulo mortis,*
And laid it on the operating table; then just as quickly brought his
Instruments, and cutting down, through one tissue, then another,
he did not stop till he had opened the trachea, *secundem artem,*
and with a pair of Gross' latest improved alligator forceps
seized the offending foreign body and hastened in triumph to
show it to the anxious waiting mother,
This fine old Irish surgeon, from Erin's emerald isle.

Och, madam, dear, here's the grain of corn,—I got it, you just bet!
Faith, it's meself's the man to do the thing to which me hand is set!
The *baby?* Oh, the baby,—(I think it was, ye said?)—
I got the murtherin' grain o' corn, I did,—but the *baby,* marm,
is dead,
Said this fine old Irish surgeon, with his most complacent smile.

